# Scavenger Receptor Class B, Type I, a CD36 Related Protein in* Macrobrachium nipponense*: Characterization, RNA Interference, and Expression Analysis with Different Dietary Lipid Sources

**DOI:** 10.1155/2016/6325927

**Published:** 2016-11-24

**Authors:** Zhili Ding, Na Luo, Youqin Kong, Jingfen Li, Yixiang Zhang, Fang Cao, Jinyun Ye

**Affiliations:** ^1^Zhejiang Provincial Key Laboratory of Aquatic Resources Conservation and Development, Key Laboratory of Aquatic Animal Genetic Breeding and Nutrition, CAFS, College of Life Science, Huzhou University, Huzhou, Zhejiang 313000, China; ^2^College of Fisheries and Life Science, Dalian Ocean University, Dalian 116000, China

## Abstract

The scavenger receptor class B, type I (SR-BI), is a member of the CD36 superfamily comprising transmembrane proteins involved in mammalian and fish lipid homeostasis regulation. We hypothesize that this receptor plays an important role in* Macrobrachium nipponense* lipid metabolism. However, little attention has been paid to SR-BI in commercial crustaceans. In the present study, we report a cDNA encoding* M. nipponense* scavenger receptor class B, type I (designated as MnSR-BI), obtained from a hepatopancreas cDNA library. The complete MnSR-BI coding sequence was 1545 bp, encoding 514 amino acid peptides. The MnSR-BI primary structure consisted of a CD36 domain that contained two transmembrane regions at the N- and C-terminals of the protein. SR-BI mRNA expression was specifically detected in muscle, gill, ovum, intestine, hepatopancreas, stomach, and ovary tissues. Furthermore, its expression in the hepatopancreas was regulated by dietary lipid sources, with prawns fed soybean and linseed oils exhibiting higher expression levels. RNAi-based SR-BI silencing resulted in the suppression of its expression in the hepatopancreas and variation in the expression of lipid metabolism-related genes. This is the first report of SR-BI in freshwater prawns and provides the basis for further studies on SR-BI in crustaceans.

## 1. Introduction

Members of the CD36 scavenger receptor protein superfamily play important roles in regulating lipid metabolism and innate immunity [1]. The superfamily is composed of SR-BI (the scavenger receptor class B, type I), LIMP2 (lysosomal integral membrane protein 2), and CD36 [1]. SR-BI, LIMP2, and CD36 are designated as scavenger receptors class B (SR-Bs), based on the differences in ligand binding specificities with class A scavenger receptors [2]. In mammals, SR-Bs have two transmembrane domains flanking an extracellular loop, with both the amino- and carboxyl-termini located in the cytoplasm [1]. Earlier work has demonstrated that SR-BI can bind to a variety of ligands, such as unmodified low density lipoproteins (LDL), very low density lipoproteins, acetylated LDL, and oxidized LDL [2]. In vitro and in vivo studies have demonstrated that SR-BI is a physiologically relevant high density lipoprotein (HDL) receptor that mediates the selective uptake of lipoprotein (HDL)-derived cholesteryl ester [3–5]. In addition to its main role of facilitating selective cholesteryl ester uptake, SR-BI also regulates processes involved in cellular cholesterol homeostasis, bidirectional cholesterol flow, membrane lipid expression, female fertility (oocyte maturation), apoptosis, and platelet function [6].

SR-BI activity can be induced in rats by PPAR*α* [7], a ligand-activated transcription factor in lipid metabolism [8]. Likewise, activation of PPAR*α* and PPAR*γ* induces SR-BI protein levels in human macrophages in vitro and in atherosclerotic lesions of Apo-E-deficient mice in vivo [9]. Therefore, fatty acids, which are natural ligands for PPAR [10], can alter SR-BI expression. Increased hepatic SR-BI mRNA and protein levels have been observed in hamsters fed polyunsaturated fatty acids [11], while treatment with saturated fatty acids reduces hepatic SR-BI gene expression [12, 13].

Some studies have also reported the structure and function of SR-Bs in invertebrates. The CD36 homolog Croquemort, a class B member of the SR family, was first described in* Drosophila melanogaster* [14]. Croquemort can act both as an essential receptor for phagocytosis of apoptotic corpses [15] and as a phagocytic receptor for Gram-positive bacteria [16]. Croquemort orthologs have also been described in* Anopheles gambiae* [17] and* Marsupenaeus japonicus* [18]. MjSR-BI, the only SR-BI identified in shrimp to date, has been reported in* M. japonica* [19]. However, these studies only focused on the SR-B's immune function, and little attention has been paid to its involvement in lipid metabolism.

*Macrobrachium nipponense* is an important prawn in China, Japan, and Southeast Asian countries because of its flavor and disease resistance. Therefore, many lipid nutrition studies and preliminary regulatory mechanisms have been performed in* M. nipponense* [20, 21]. Considering the many functions of SR-BI, particularly its role in lipid homeostasis, we hypothesize that the receptor's expression is regulated by dietary lipid composition in* M. nipponense*. In this paper, a complete SR-BI coding sequence (cds) was obtained from the* M. nipponense* hepatopancreas transcriptome (NCBI GEO accession number: GSE78788). Its structural characteristics and mRNA expression patterns in different tissues were analyzed. We also analyzed the mRNA expressions of SR-BI and other lipid metabolism-related genes (fatty acid-binding protein 10 [FABP10], acyl-CoA binding protein [ACBP], carnitine palmitoyltransferase-1 [CPT-1], and acetyl-CoA carboxylase [ACC]) after SR-BI dsRNA injection in* M. nipponense* fed different sources of dietary lipids.

## 2. Materials and Methods

### 2.1. Experimental Animals, Feeding Trial, and Sample Preparation

Healthy juvenile prawns (0.124 ± 0.004 g) were randomly stocked in twenty 300 L tanks with 50 prawns per tank (five replicates per dietary group). Six semipurified diets with different lipid sources were formulated to feed the prawns. The six lipid sources were medium chain triglyceride (MCT) oil, lard oil (LO), soybean oil (SO), linseed oil (LIO), pollock fish oil (FO), and a mixture of fish and soybean oil (FO/SO 2 : 1 w/w). The formulation process was the same as previously described [22]. Fatty acid compositions of the diets were analyzed by gas chromatography (Hewlett-Packard Model HP 5890, CA, USA) as described previously [23], and the ingredients and fatty acid composition of these diets are shown in Tables 1 and 2, respectively. The prawns were exposed to a natural photoperiod and fed to apparent satiation twice a day for 56 days. Temperature, dissolved oxygen, and ammonia-nitrate were 27–29°C, >6.5 mg L^−1^, and <0.1 mg L^−1^, respectively. At the end of the feeding trial, all prawns were counted and survival rate [survival rate = 100 × (final prawn number)/(initial prawn number)] was obtained. And the hepatopancreases of six treatment groups were dissected from the cephalothorax and the abdomen and stored at −80°C for RNA extraction and analysis.

Healthy oriental river prawns* M. nipponense *were obtained from the aquatic product market, Huzhou, China. Tissues were collected from the prawns after having been maintained in aerated freshwater for 72 h. A variety of tissues including muscle, gill, ovum, ovary, intestine, ovary, hepatopancreas, heart, stomach, and hemocyte were collected and stored at −80°C for further study.

### 2.2. RNA Extraction and Reverse Transcription

RNA was extracted with an RNA extraction kit (Aidlab Biotech, Beijing, China) following the manufacturer's protocol. In the kit, there was a column to remove the genomic DNA, and the quality and quantity of the total RNA were determined by using a NanoDrop 1000 spectrophotometer (Hach, America). The cDNA was synthesized from 5 *μ*g of total RNA using the PrimeScript™ RT-PCR Kit (Takara, Japan) according to the manufacturer's instructions. The cDNA was kept at −20°C for real-time quantitative RT-PCR (qRT-PCR).

### 2.3. SR-BI Sequence Analysis

Based on the homology to* Marsupenaeus japonicus* and other organisms, we identified the complete SR-BI cds in the* M. nipponense* hepatopancreas and muscle transcriptome libraries (NCBI GEO accession number GSE78788). The two primers SR-BI-F and SR-BI-R validated the SR-BI sequence from the cDNA library.

The searches for protein sequence similarities were conducted with the BLAST algorithm at the National Center for Biotechnology Information (http://www.ncbi.nlm.nih.gov/BLAST/). The protein prediction was performed in the ORF finder (http://www.ncbi.nlm.nih.gov/gorf/). Both the theoretical molecular mass and isoelectric point were predicted through a public website (http://us.expasy.org/tools). Signal sequence and motif prediction were carried out in SMART (http://smart.embl-heidelberg.de/).* M. nipponense* SPH deduced amino acid sequences and class B scavenger receptor amino acid sequences from other species were compared by multiple sequence alignment in ClustalX. A bootstrap neighbor-joining (NJ) phylogenetic tree was constructed with the amino acid sequences from members of the CD36 scavenger receptor protein superfamily retrieved from the NCBI website using MEGA version 4.0 (http://www.megasoftware.net/).

### 2.4. Analysis of SR-BI Gene Expression

SR-BI mRNA expression in different tissues was detected by SYBR® Green real-time quantitative RT-PCR (qRT-PCR). The first-strand cDNA preparation was as described above. A predicted SR-BI PCR amplicon of 186 bp was generated using the gene-specific primer pair SR-BI-F1 and SR-BI-R1 (Table 1). The primers*β*-actin-F and*β*-actin-R (Table 1) were used to amplify the 247 bp fragment that served as an internal control gene [24].

The SYBR Premix Ex Taq™ Kit (Takara) was used for real-time quantitative RT-PCR (qRT-PCR) analysis in a CFX96™ Real-Time System (Bio-Rad, USA). Amplifications were performed on a 96-well plate with a 20 *μ*L reaction volume containing 10 *μ*L 2x SYBR Green Premix Ex Taq, 0.2 *μ*L of each primer (10 *μ*M), 2 *μ*L template, and 7.6 *μ*L PCR grade water. The PCR temperature profile was as follows: 95°C for 30 s followed by 40 cycles of 94°C for 15 s, 58°C for 20 s, and 72°C for 20 s, with a 0.5°C/5 s incremental increase from 60 to 95°C. 2^−ΔΔCt^ comparative CT method [25] was used to analyze the expression level of these genes, where*β-*actin was used as reference gene to normalize the expression data.

### 2.5. dsRNA Synthesis and Injection

Either MnSR-BI dsRNA or green fluorescent protein (GFP) was synthesized in vitro with a Transcript Aid™ T7 High Yield Transcription Kit (Thermo Scientific, Inc., Massachusetts, USA) according to the manufacturer's instructions. Briefly, a PCR fragment containing an MnSR-BI (343 bp) open-reading frame was amplified using gene-specific primers. The 280 bp GFP control fragment was produced by PCR of the pEGFP-C1 plasmid.

The MnSR-BI and GFP primers containing a T7 promoter site at the 5′ ends of the gene-specific primers are shown in Table 3. To generate dsRNA, PCR products purified by gel extraction (Sangon, Shanghai, China) were used as templates for in vitro transcription with a Transcript Aid T7 High Yield Transcription Kit. The dsRNA was purified by ethanol precipitation and dissolved in RNase-free water. dsRNA purity and integrity were determined by standard agarose gel electrophoresis. The dsRNA concentration was measured at 260 nm using a BioPhotometer (Eppendorf, Hamburg, Germany) and then kept at −20°C until required. For the dsRNA injection test, there were two treatments in every diet group. Briefly, 100 *μ*g of MnSR-BI or GFP (control) dsRNA was dissolved in 100 *μ*L saline (0.87% (w/v), pH 7.0). Individual prawns were injected with either 10 *μ*g MnSR-BI (*n* = 10) or GFP (*n* = 10) dsRNA from the* M. nipponense* abdominal body cavity. The injection time was performed at the end of the feeding trial.

### 2.6. SR-BI and Related Gene Expression after dsRNA Injection

**Figure 1:**
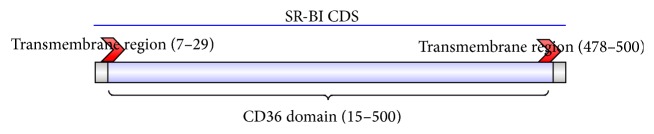
A schematic representation of the MnSR-BI protein complete CDS (514 amino acids), the blue part representing the CD36 domain, and the two transmembrane regions were at the N- and C-terminals of the protein.

At 48 and 96 h after dsRNA injection, the prawn hepatopancreases were dissected and stored at −80°C for RNA extraction and analysis. The SR-BI mRNA expression in both treatments in every diet group was detected by qRT-PCR 48 and 96 h after dsRNA injection. SR-BI can bind to a variety of ligands, such as LDL, acetylated LDL, oxidized LDL, and HDL [2–5]. The SR-BI of* M. nipponense* had a CD36 domain (Figure 1). As we all know, CD36 plays a role in long chain fatty acid transport and uptake in mitochondria [26]. Furthermore, no CD36 nucleotide was present in the* M. nipponense* hepatopancreas and muscle transcriptome libraries (NCBI GEO accession number GSE78788). So we hypothesize that SR-BI of* M. nipponense* is a multifunctional protein and may have an important role in fatty acid transport. If SR-BI was silenced, the genes involved in fatty acid*β*-oxidation and fatty acid biosynthesis may take some change, the same as genes involved in intracellular transport of fatty acids. In this paper, Cpt-1 and ACC are the key genes involved in fatty acid*β*-oxidation and fatty acid biosynthesis, respectively [27, 28]. FABP10 and ACBP are related to intracellular fatty acids metabolism, which can bind intracellular fatty acid and acyl-CoAs, respectively [29, 30]. Therefore, the related lipid metabolism genes (FABP10, ACBP, CPT-1, and ACC) were also detected by qRT-PCR 48 h after dsRNA injection. Gene-specific primers were designed with Primer3 (http://primer3.ut.ee/) [31], based on the cDNA sequences in GenBank (FABP10: JN995589; ACBP: KF896234; CPT-1: KP690136; ACC: KP690138) (Table 3).

### 2.7. Statistical Analysis

Statistical analysis was performed in SPSS (Version 17.0, IBM SPSS, Chicago, IL, USA). All data are given as means ± standard deviation (SD). The results of relative SR-BI gene mRNA expression and related lipid metabolism gene expression after dsRNA injection were subjected to* t*-tests, while the other results were determined by one-way ANOVA and post hoc Tukey's multiple comparisons. *P* values < 0.05 were considered significant.

## 3. Results

### 3.1. Molecular Characterization and Phylogenetic Analysis of SR-BI

**Figure 2:**
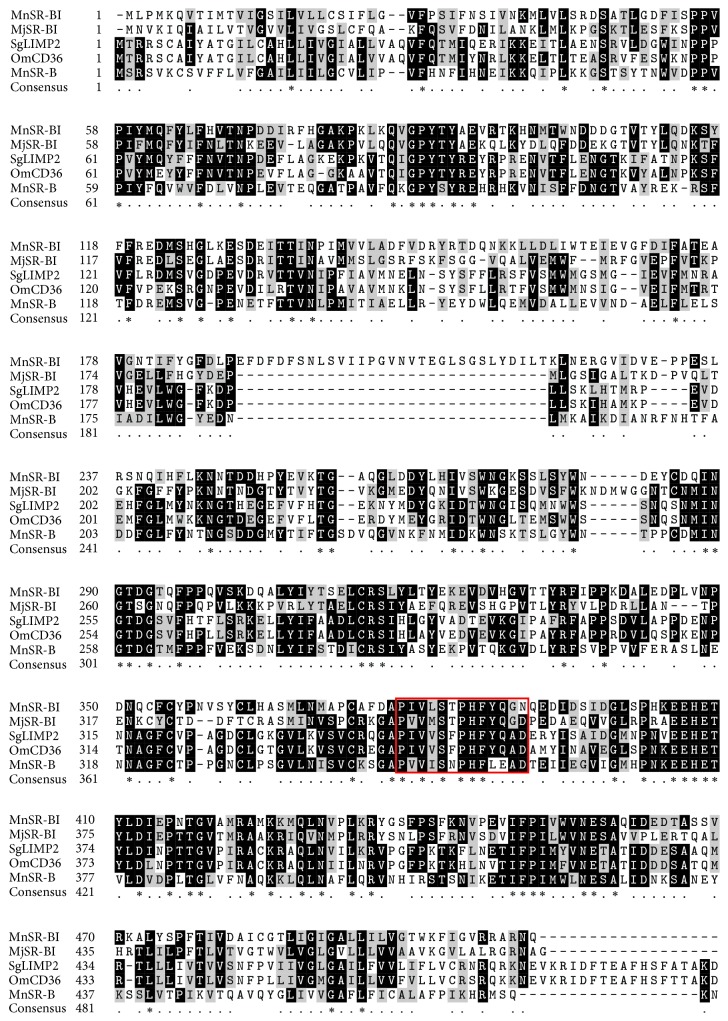
Multiple alignments of class B scavenger receptors. Species names are abbreviated on the left and represent* Macrobrachium nipponense* scavenger receptor class B, type I (MnSR-BI) [ALK82306];* Marsupenaeus japonicus* scavenger receptor class B, type I (MjSR-BI) [AKO62849.1];* Sinocyclocheilus grahami* lysosome membrane protein 2- (SgLIMP2-) like [XP_016108260.1];* Oncorhynchus mykiss* CD36 antigen (OmCD36) [NP_001117983.1]; and* Mimachlamys nobilis* scavenger receptor class B- (MnSR-B-) like protein [AJM13625.1]. Identical residues are shaded black, while conserved groups are gray. A 13-amino acid motif present in members of the CD36 superfamily is highlighted by the red box.

The complete MnSR-BI coding sequence (cds) was 1545 bp (KP690136). Analysis of the deduced MnSR-BI protein sequence revealed that it was composed of 514 amino acids with a predicted molecular mass of 58.22 kDa and an isoelectric point of 4.81. The MnSR-BI had a CD36 domain that contained two transmembrane regions at the N- (started at position 7 and ended at position 29) and C-terminals (started at position 478 and ended at position 500) of the protein (Figure 1). The NCBI BLASTP program revealed that the predicted MnSR-BI amino acid sequence exhibited 41% identity with* M. japonicus*  SR-BI (AKO62849.1), 35% with an SR-B-like protein in* Mimachlamys nobilis* (AJM13625.1), 35% with* Sinocyclocheilus grahami* LIMP (XP_016108260.1), and 34% with the* Oncorhynchus mykiss* CD36 antigen (NP_001117983.1). A 13 aa's (amino acids) motif that is conserved in these proteins “P I/V Y I/L S F/L P H F Y/L L A D/S” was identified (Figure 2).

**Figure 3:**
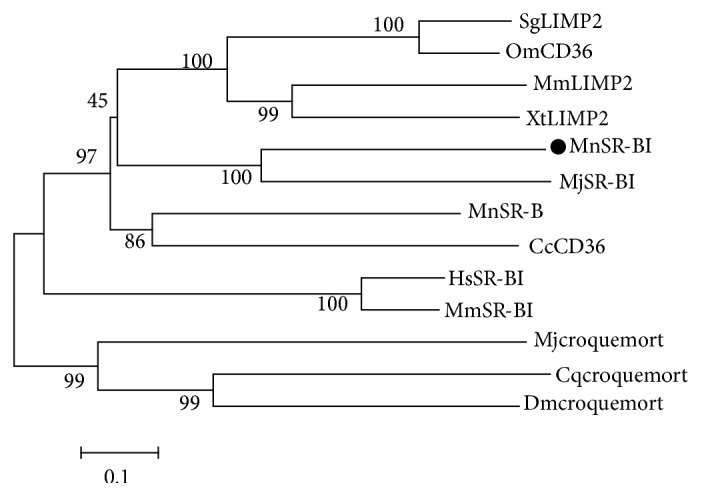
Phylogenetic tree inferred from arthropod PPAFs amino acid sequences. Species names are abbreviated in the tree to represent* Culex quinquefasciatus* Croquemort (Cqcroquemort) [XP_001844488];* Cyprinus carpio* CD36 (CcCD36) [AIT69834];* Drosophila melanogaster* Croquemort (Dmcroquemort) [NP_787957];* Homo sapiens* scavenger receptor class B member 1 (HsSR-BI) isoform [NP_005496];* Macrobrachium nipponense* scavenger receptor class B, type I (MnSR-BI) [ALK82306];* Marsupenaeus japonicus* croquemort (Mjcroquemort) [BAJ10664];* Marsupenaeus japonicus* scavenger receptor class B (MjSR-BI) [AKO62849];* Mimachlamys nobilis* scavenger receptor class B (MnSR-B-) like protein [AJM13625];* Mus musculus* scavenger receptor class B member 1 (MmSR-BI) isoform 1 [NP_058021];* Mus musculus* lysosome membrane protein 2 (MmLIMP2) [NP_031670];* Oncorhynchus mykiss* CD36 antigen (OmCD36) [NP_001117983];* Sinocyclocheilus grahami *lysosome membrane protein 2- (SgLIMP2-) like [XP_016108260];* Xenopus tropicalis* lysosome membrane protein 2 (XtLIMP2) [NP_001016557].

A phylogenetic tree was constructed based on the NJ analysis (Figure 3). Phylogenetic analysis of available scavenger receptor amino acid sequences revealed that the SR-Bs divided into two groups. Scavenger receptor class B, type I, LIMP2, and CD36 formed one group, while the scavenger receptor Croquemort formed another. MnSR-BI was more closely related to* M. japonicus*  SR-BI.

### 3.2. SR-BI mRNA Distribution in Tissues

**Figure 4:**
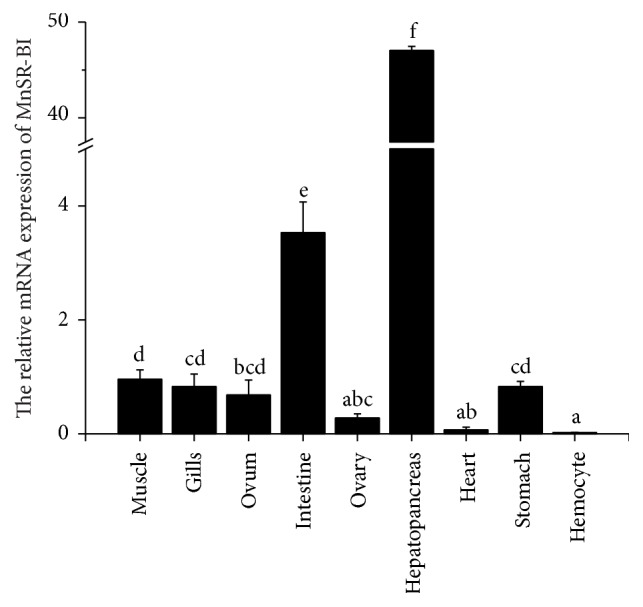
MnSR-BI expression tissue distribution as determined by real-time qRT-PCR. Values are shown as mean ± SD (*n* = 3). Bars with different letters represent significant differences (*P* < 0.05).

The MnSR-BI tissue distribution pattern was analyzed by qRT-PCR, which revealed that it was predominantly expressed in the muscle, gill, ovum, intestine, hepatopancreas, stomach, and ovary tissues (Figure 4). MnFABP expression was very low in the hemocytes and heart. However, its expression was significantly higher in the hepatopancreas than in other tissues, being almost 49.17- and 13.32-fold greater compared with that in the muscle and intestine, respectively.

### 3.3. SR-BI Expression in Response to Dietary Lipid Sources

**Figure 5:**
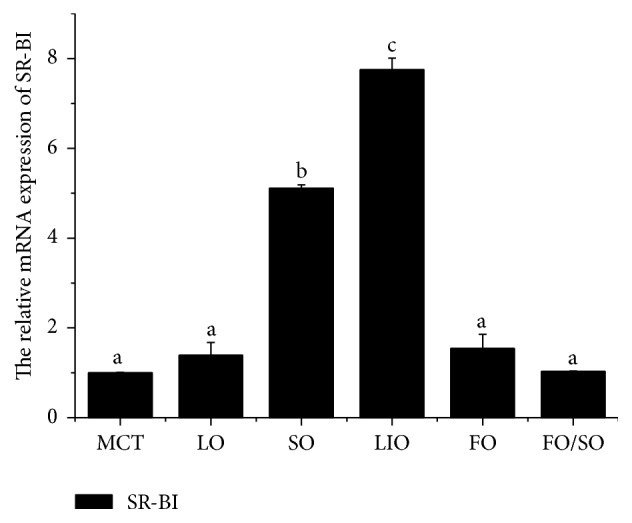
MnSR-BI mRNA expression in the hepatopancreas of* M. nipponense* fed different dietary lipid sources for 56 days. Bars represent mean ± SD (*n* = 3). Bars with different letters differ significantly (*P* < 0.05).

Prawn survival rate (72%–80%) was not affected by the dietary lipid sources. The prawns fed LIO and SO had higher MnSR-BI expression levels in the hepatopancreas than the prawns fed other diets (*P* < 0.05). No significant difference in MnSR-BI expression in the hepatopancreas was observed among the MCT, LO, FO, and FO/SO groups (Figure 5).

### 3.4. SR-BI and Other Lipid Metabolism-Related Gene Expressions after dsRNA Injection

**Figure 6:**
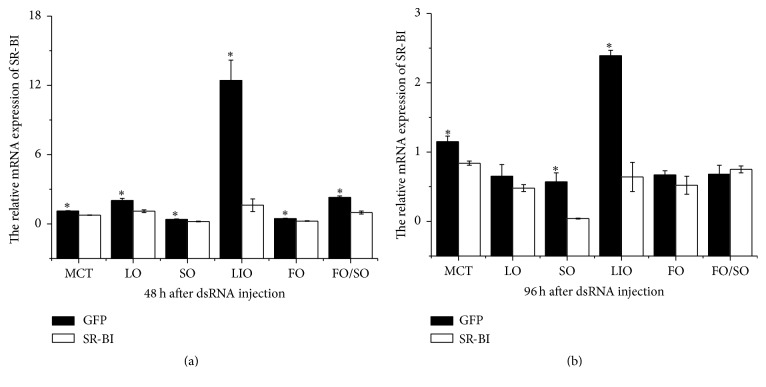
Real-time quantitative PCR analysis of the MnSR-BI transcript expression in the hepatopancreas of* M. nipponense* injected with either MnSR-BI or GFP dsRNA. Samples were obtained 48 h (a) and 96 h (b) after dsRNA injection and analyzed by real-time qRT-PCR. Bars indicate mean ± SD (*n* = 3). ^*∗*^ *P* < 0.05.

The MnSR-BI gene was examined by real-time qRT-PCR to determine the validity of RNA interference (Figure 6). MnSR-BI expression in prawns injected with MnSR-BI dsRNA at 48 h decreased significantly compared with that of prawns injected with GFP dsRNA (*P* < 0.05). MnSR-BI expression in prawns from the MCT, SO, and LIO groups injected with MnSR-BI dsRNA at 96 h was also significantly higher than that of prawns injected with GFP dsRNA. No significant difference in MnSR-BI expression was observed between MnSR-BI and GFP dsRNA in the LO, FO, and FO/SO diet groups at 96 h.

**Figure 7:**
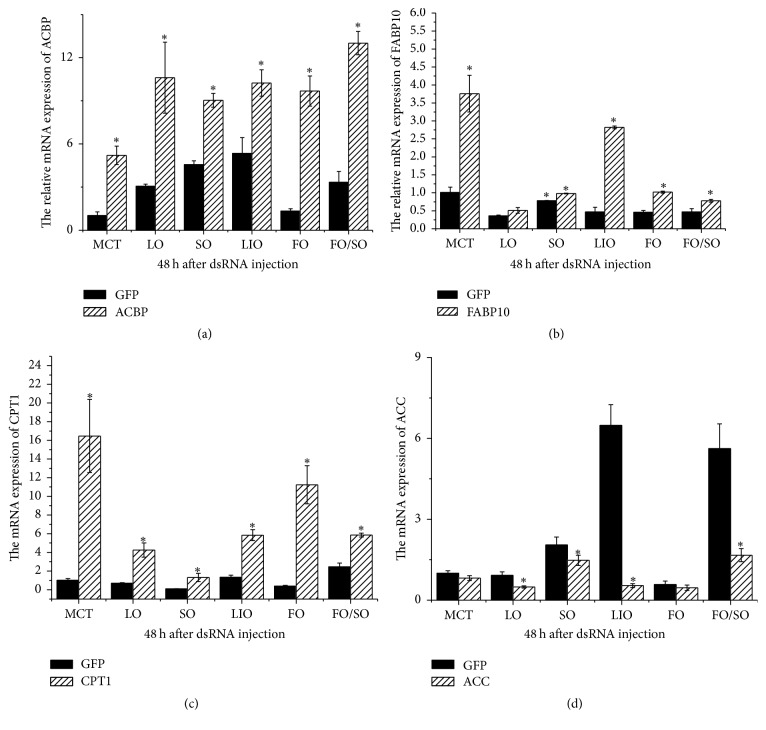
Real-time quantitative PCR analysis of ACBP (a), FABP10 (b), CPT1 (c), and ACC (d) transcript expressions in the hepatopancreas of* M. nipponense* injected with either MnSR-BI or GFP dsRNA at 48 h. Bars indicate mean ± SD (*n* = 3). ^*∗*^ *P* < 0.05.

CPT1 mRNA expression in prawns injected with MnSR-BI dsRNA at 48 h increased significantly compared to that of prawns injected with GFP dsRNA in the six dietary lipid sources treatments (*P* < 0.05) (Figure 7). Similar results were observed for ACBP mRNA expression in prawns from all groups. FABP10 expression in prawns injected with MnSR-BI dsRNA at 48 h was also significantly higher than that of prawns injected with GFP dsRNA, except for the LO group (*P* < 0.05). However, ACC expression in prawns on the LO, SO, LIO, and FO/SO diets injected with MnSR-BI dsRNA at 48 h decreased significantly compared to the control (*P* < 0.05), and no significant difference was observed between the MCT and FO diet treatments.

## 4. Discussion

In this study, we characterized the structure and expression profile of an SR-BI from* M. nipponense*. Structurally, MnSR-BI has a CD36 domain that contains two transmembrane regions at the N- and C-terminals of the protein, which is consistent with* M. japonica* SR-BI [19]. The scavenger receptor transmembrane proteins participate in the recognition of a broad range of polyanionic ligands, including modified and oxidized low density lipoproteins, bacteria, and apoptotic cells [32]. The ClustalX alignment of MnSR-BI and other SR-B sequences revealed relatively low conservation in the SR-B family (34–41%). Of particular interest was the 13 aa's (amino acids) motif in these proteins “P I/V Y I/L S F/L P H F Y/L L A D/S,” which is conserved among all the members of the CD36 superfamily [33]. Phylogenetic analysis of SR-Bs from representative animals produced the NJ-phylogenetic tree containing two distinct branches. Scavenger receptor class B, type I, LIMP2, and CD36 formed one branch, while the scavenger receptor Croquemort formed the other. MnSR-BI and* M. japonicus*  SR-BI exhibited the same branch pattern, indicating that of the species examined MnSR-BI is the closest to the* M. japonicus*  SR-BI. The conserved domain and similarity with other SR-Bs suggest that MnSR-BI is a member of the CD36 scavenger receptor superfamily.

By means of qRT-PCR analysis, MnSR-BI transcripts were detected in most of the organs examined, while traces were detected in the hemocytes and heart. The strong expression observed in the hepatopancreas was similar to that reported in rat studies [34, 35]. In the liver, SR-BI is mainly expressed in parenchymal cells (hepatocytes), which account for >90% of liver mass [35]. Thus, the liver expresses the highest amount of SR-BI on an organ basis [36]. Interestingly, numerous reports have shown abundant SR-BI expression in mammalian ovaries [34, 37]. In the present study, we also observed SR-BI expression in the* M. nipponense* ovum and ovary. Krieger [38] suggests that SR-BI is expressed at high levels by steroidogenic tissues and the liver, in which it seems to have important functions in selective uptake of cholesterol from HDL and in mediating reverse cholesterol transport. As we all know, the hepatopancreas is the main lipid storage and processing organ in crustaceans [39]. So it is not surprising that the SR-BI is mainly expressed in hepatopancreas of this prawn.

In vertebrates, the scavenger receptor CD36 domain binds to oxidized LDL, modified LDL, long chain fatty acids, and anionic phospholipids [40]. The presence of a CD36 domain in MnSR-BI suggests that it could be acting as a scavenger receptor for either one or several of these molecules [18]. In this study we used qRT-PCR to examine the nutritional regulation of SR-BI. The prawns fed SO and LIO had increased SR-BI expression levels compared with prawns fed other diets, which suggests that dietary fatty acids play a major role in regulating hepatopancreatic MnSR-BI expression in* M. nipponense*. It has been demonstrated that dietary fatty acids can regulate plasma lipoprotein concentrations [41]. High levels of polyunsaturated fatty acids may also lower plasma HDL cholesterol concentrations [42, 43]. Considering the main role of SR-BI in regulating cellular cholesterol concentrations, SO and LIO, which are rich in 18C polyunsaturated fatty acids (linoleic acid and *α*-linolenic acid, resp.), might have a stronger effect on hepatopancreatic cholesteryl ester uptake than other saturated (MCT and LO) and n-3 highly unsaturated fatty acids (FO and FO/SO) in the* M. nipponense* hepatopancreas. Spady et al. [11] have also shown that, in hamsters, a diet rich in polyunsaturated fatty acids increases SR-BI expression compared to a diet rich in saturated fatty acids. The SR-BI activity is induced by the PPAR*α* in rats [7]. Fatty acids are natural ligands for PPAR [10], which may activate PPAR*α* and induce SR-BI expression. However, the exact mechanism whereby dietary fatty acids regulate hepatopancreatic SR-BI expression is unknown. Loison et al. [12] demonstrated that myristic acid could regulate HDL cholesterol via SR-BI by a mechanism that implicates either SREBP or other transcription factors in the hamster.

RNAi-based silencing of MnSR-BI resulted in a significant decrease in MnSR-BI compared with that of prawns injected with GFP dsRNA at 48 h. Thereafter, an increase in CPT1, FABP10, and ACBP mRNA expression was observed. Carnitine palmitoyltransferase 1 (CPT1) is considered the key regulatory enzyme in mitochondrial fatty acid *β*-oxidation [27]. FABPs are involved in intracellular fatty acid uptake and transport [29]. The basic liver-type FABP (Lb-FABP or FABP10) was one of the FABPs first isolated from chicken liver [44]. ACBP can maintain intracellular acyl-CoA pool size and transport acyl-CoAs between different subcellular membranes [30]. The increase in CPT1, FABP10, and ACBP mRNA expression suggests that upregulation of mitochondrial fatty acid oxidation and fatty acid transport and uptake might compensate for decreased SR-BI expression. ACC is a rate-limiting enzyme that catalyzes the carboxylation of acetyl-CoA to form malonyl-CoA, the first step in the long chain fatty acid biosynthesis pathway [28]. Conversely, we saw a significant decrease in ACC expression in the* M. nipponense* hepatopancreas. Based on the present results, it seems that there is an association between SR-BI and CPT1, FABP10, ACBP, and ACC expression. Whether there are signaling mechanisms upstream or downstream of SR-BI affecting the expression of lipid metabolism-related genes in* M. nipponense* requires further investigation.

## Figures and Tables

**Table 1:** Ingredient composition and nutrient content of the test diets (%).

Ingredients (%)	Test diets					
	MCT	LO	SO	LIO	FO	FO/SO
Casein	30	30	30	30	30	30
Fish meal	20	20	20	20	20	20
Corn starch	25	25	25	25	25	25
^a^Lipids	6	6	6	6	6	6
Attractant	3	3	3	3	3	3
Soybean lecithin	0.5	0.5	0.5	0.5	0.5	0.5
Choline chloride	0.5	0.5	0.5	0.5	0.5	0.5
^b^Mineral mixture	3	3	3	3	3	3
^c^Vitamin mixture	2	2	2	2	2	2
Cellulose	8	8	8	8	8	8
Sodium carboxymethylcellulose	2	8	8	8	8	8
Proximate composition (air dry matter)						
Crude protein	40.12	40.02	39.92	39.75	40.05	39.89
Crude lipid	8.21	8.23	8.34	8.25	8.36	8.12

^a^ Six oil sources including medium chain triglyceride oil (MCT), lard oil (LO), soybean oil (SO), linseed oil (LIO), fish oil (FO), or a mixture of 2 to 1 ratios of fish oil and soybean oil (FO/SO) were used.

^b^ Vitamin mixture (100 g^−1^ mixture): vitamin A, 420000 IU; vitamin C, 6000 mg; *α*-tocopherol acetate, 2000 mg; vitamin D3, 120000 IU; vitamin K, 1000 mg; vitamin B1, 1000 mg; vitamin B2, 1000 mg; vitamin B6, 1600 mg; vitamin B12, 2 mg; niacin, 5000 mg; folic acid, 400 mg; inositol, 6000 mg; biotin, 10 mg; calcium pantothenic, 3500 mg.

^c^ mineral mixture (mg g^−1^ mixture): KCL, 28; MgSO_4_·7H_2_O, 100; NaH_2_PO_4_, 215; KH_2_PO_4_ 100; Ca(H_2_PO_4_)_2_·H_2_O, 265; CaCO_3_, 105; C_6_H_10_CaO_6_·5H_2_O, 165; FeC_6_H_5_O_7_·5H_2_O, 12; ZnSO_4_·7H_2_O, 4.76; MnSO_4_·H_2_O, 1.07; AlCL_3_·6H_2_O, 0.15; CuCl_2_·2H_2_O, 0.24; CoCl_2_·6H_2_O, 1.4; KI,0.23; Na_2_SeO_3_ 0.0009.

**Table 2:** Main fatty acid composition (% of total fatty acids) of experimental diets.

Fatty acids	Test diets					
	MCT	LO	SO	LIO	FO	FO/SO
8:0	34.65	0.15	—	—	—	—
10:0	43.45	37.17	—	—	—	—
14:0	1.25	1.22	0.58	0.73	4.73	3.17
16:0	7.69	22.07	12.69	9.63	19.62	17.04
18:0	2.29	14.03	5.28	6.29	5.38	5.28
∑SFA	82.54	38.45	19.61	17.41	31.95	27.34
16:1n-7	1.28	1.88	—	0.82	5.45	3.77
18:1n-9	5.93	38.89	22.36	19.39	17.25	18.91
∑MUFA	7.65	42.09	22.91	20.33	26.34	25.15
18:2n-6	2.79	13.47	45.32	14.05	12.04	23.82
20:4n-6	0.40	0.47	0.28	0.29	1.15	0.80
∑n-6 PUFA	3.19	14.02	45.94	14.34	13.42	24.90
18:3n-3	0.67	0.83	6.60	43.45	5.27	5.14
20:5n-3	1.67	1.22	1.21	1.28	9.30	6.82
22:6n-3	3.83	2.85	2.69	2.83	12.47	9.42
∑n-3 PUFA	6.53	5.32	10.72	47.98	28.02	22.40

Data are mean of duplicate assay. Only the major fatty acids are shown in the table, and the detected fatty acids include C14:0, C15:0, C16:0, C17:0, C18:0, C14:1, C16:1, C17:1, C18:1n-9, C20:1n-9, C22:1n-9, C24:1n-9; C20:2, C22:2, C18:2n-6, C18:3n-3, C18:3n-6, C20:4n-6, C20:5n-3, C22:5n-3, and C22:6n-3. ∑SFA is the sum of saturated fatty acids. ∑MUFA is the sum of monounsaturated fatty acids. ∑PUFA is the sum of polyunsaturated fatty acids. ∑n-6 PUFA is the sum of n-6 polyunsaturated fatty acids. ∑n-3 PUFA is the sum of n-3 polyunsaturated fatty acids.

**Table 3:** Primers used in this study.

Name	Sequence (5′-3′)
MnSR-BI-F	GAGAAAGAGGTAGATGTCCAC
MnSR-BI-R	CGTGAATGGAGAATAGAGAGC
MnSR-BI-F1	TGCAGTTCTACCTCTTTCAC
MnSR-BI-R1	TGTCCTCCCTGAAGAAGTAA
dsMnSR-BI-F	TAATACGTCACTATAGGG ACTTTGCAGTATCTTCCTGG
dsMnSR-BI-R	TAATACGTCACTATAGGG GATCTCATCAGACTCCTTCAG
dsGFP-F	TAATACGTCACTATAGGG CACATGAAGCAGCACGACTTC
dsGFP-R	TAATACGTCACTATAGGG TGTGGCGGATCTTGAAGTTCA
FABP10-F	CCAAGCCAACTCTGGAAGTC
FABP10-R	GATCTCAACGCTGGCTTCTC
ACBP-F	CCTAATGATGAGGAGCTG
ACBP-R	GTTGCAATCTCCTACAGTT
CPT1-F	AATTTTTGACTGGCTTCTCC
CPT1-R	TCCATTCTGGAAATCATCTG
ACC-F	CAAGGTCCACTACATGGTCT
ACC-R	ACTCTTCCCAAACTCTCTCC
*β*-Actin-F	GTGCCCATCTACGAGGGTTA
*β*-Actin-R	CGTCAGGGAGCTCGTAAGAC
